# Is the bell ringing for another outbreak of Crimean-Congo hemorrhagic fever in Pakistan?

**DOI:** 10.1016/j.puhip.2022.100319

**Published:** 2022-09-19

**Authors:** Abdul Waris, Faheem Anwar, Muhammad Asim, Farkhanda Bibi

**Affiliations:** Department of Biomedical Sciences, City University of Hong Kong, Hong Kong Special Administrative Region; Department of Biotechnology and Genetic Engineering, Hazara University Mansehra, Pakistan; Department of Biomedical Sciences, City University of Hong Kong, Hong Kong Special Administrative Region; Faculty of Biological Sciences, Abdul Wali Khan University Mardan, Pakistan

**Keywords:** CCHF, Re-emerging virus, Outbreak, Pakistan

Dear Editor,

Crimean-Congo hemorrhagic fever (CCHF) is a fatal viral infection caused by Crimean-Congo hemorrhagic Fever Virus (CCHFV), with a case fatality rate of 10–40% [1]. The CCHFV belongs to the genus Nairovirus within the family Bunyaviridae and is considered the world's most prevalent tick-borne virus. CCHFV can be transmitted to humans through contact with blood or tissues of the infected animal immediately during or after slaughter or through direct tick bites. In contrast, human-to-human transmission occurs through direct contact with the infected person's blood, organs, secretions, or any other fluid of the infected individual. To date, no vaccines are available against CCHFV [2,3].

Pakistan is being hit with a quadruple burden of various diseases that comprises communicable and non-communicable, including the ongoing deadly pandemic of the coronavirus disease 2019 (Covid-19) [3]. In this context, CCHF is a fatal infection that needs utmost attention due to its high case fatality and mortality rate. The first case of CCHF in Pakistan was reported in 1976 in Rawalpindi, Punjab province of Pakistan, and after that, sporadic cases continued to occur in different regions of the country for different years [4]. After the first case, in the next 34 years (1976–2010), 14 cases were reported in various parts of the country, especially the rural areas where the herding of cattle is a common profession. Since 2010, CCHF has been endemic in Pakistan and increased prevalence of the disease has been occurred such as a total of 62 confirmed cases with 18 mortalities has been reported in 2012, 100 cases in 2013, 34 in 2014, 25 in 2015, 27 in 2019, 51 in 2017–18, 20 in 2019, 356 in 2020, and 13 in 2022 as of June 2022 [5].

From the clinical data, it has been reported that the majority of CCHF cases have been reported from the rural areas of Khyber Pakhtunkhwa and Baluchistan provinces of Pakistan, where the common profession of the majority of people is the herding cattle. In these rural areas, preventive measures are less common due to illiteracy, poverty, poor infrastructure, and unavailability of health and live-stock-related resources [3,6]. In 2022, Pakistan reported the first case of CCHF in Peshawar in mid-June, and to date, a total of 13 confirmed cases have been reported from various parts of the country, as shown in Fig. 1. Among all provinces, Baluchistan was always the most affected province and reported 14 positive cases of CCHF and 5 deaths in 2021. From this, it can be predicted that if no preventive measures are taken to control the spread of the virus and treat the infected people, the number of cases will be increased and will cause an alarming situation that may lead to another outbreak. Therefore, the rural areas of Pakistan, especially Baluchistan, needed special attention. The government should provide necessary facilities to the cattle handlers and educate the people about the CCHF and other related preventive measures and protective support.

**Fig. 1:**
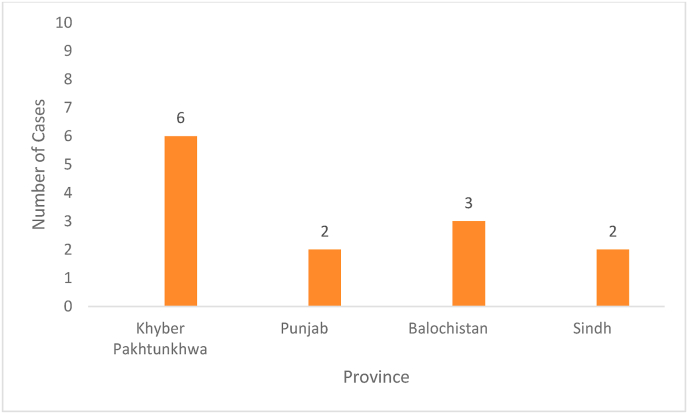
Current situation of CCHF positive cases in Pakistan.

In addition, preventive measures should be taken during Eid ul Adha; a religious festival celebrated each year on day 10 of month 12 of the Islamic year. On this day, about 5 million animals are sacrificed across the country [6]. The control of CCHF is difficult on this religious occasion because of the trade of animals and uncontrolled movement within the country. Most of the animals, such as cows, goats, sheep, camels, etc., are shifting to cities for slaughtering and sacrificing from rural areas of the country, increasing the spread of the disease. Therefore, animal checking for infection should be conducted, and the movement of animals should be strictly controlled in disease-endemic areas. The transmission of the CCHF may increase in the coming Eid Ul Adha if the policies are not revised regarding slaughter zones and checking points, testing of animals, and control movement of the animals across the country.

The diagnosis, management, and treatment of CCHFV-infected individuals should be improved, especially for people with a high risk of infection, i.e., men and women working in slaughterhouses, the agriculture sector, and health care setup. People should be educated about the issues related to CCHF. The print and electronic media and other non-government organizations should also play an important role in the awareness of people by arranging seminars, workshops, and other awareness campaigns regarding the spread and control of the disease.

## Declarations

### Ethical approval

Not Applicable.

### Competing interests

The authors declare no conflict of interest.

### Authors' contributions

All authors contributed equally.

### Funding

No funding received for this study.

### Availability of data and materials

All data generated or analysed during this study are included in this published article.
